# Very low HIV positivity on paediatric surgical wards in Mozambique: Implications for inpatient provider-initiated testing programmes

**DOI:** 10.4102/sajhivmed.v25i1.1544

**Published:** 2024-01-08

**Authors:** Henriques Viola, Angela Bi, Dalva Khosa, Zacarias Mateus, Massada da Rocha, Vanda Amado, Atanásio Taela, Daniel A. DeUgarte, Andreas Schindele, W. Chris Buck

**Affiliations:** 1 Department of Surgery, Hospital Central de Maputo, Maputo, Mozambique; 2 David Geffen School of Medicine, University of California Los Angeles, Los Angeles, United States of America; 3 Department of Surgery, Hospital Central de Nampula, Nampula, Mozambique; 4 Department of Surgery, Hospital Central da Beira, Beira, Mozambique; 5 Department of Surgery, David Geffen School of Medicine, University of California Los Angeles, Los Angeles, United States of America; 6 Department of Global Child Health, Faculty of Health, University of Witten/Herdecke, Witten, Germany; 7 Department of Pediatrics, David Geffen School of Medicine, University of California Los Angeles, Los Angeles, United States of America

## Introduction

To close diagnostic gaps for children living with HIV, the World Health Organization (WHO) recommends routine opt-out provider-initiated testing and counselling (PITC) for inpatient wards in high-prevalence countries, which includes nucleic acid testing as part of Early Infant Diagnosis (EID) programmes for HIV-exposed infants < 18 months of age.^1^ There is strong evidence of the effectiveness of paediatric inpatient PITC in improving diagnosis of HIV and linkage to antiretroviral treatment (ART) in sub-Saharan Africa (SSA), including two systematic reviews of paediatric HIV testing that reported the highest positivity rates in hospitalised children.^2,3^

However, the implementation of routine PITC remains a challenge in the SSA hospital setting and studies have reported barriers, including insufficient staff, lack of training, stock-outs of test kits, and inadequate tools for monitoring and evaluation.^4,5,6^ Inpatient PITC is particularly challenging in paediatrics, as screening for breastfeeding infants requires testing their mothers, and many hospitals lack access to timely EID nucleic acid testing.^1^ As a result, testing coverage rates are low and many children are discharged without confirmation of HIV status.^7^

The testing yield from paediatric inpatient PITC has declined over the last 20 years, with advances in prevention of mother-to-child transmission (PMTCT) programmes.^8^ An analysis of the United States President’s Emergency Plan for AIDS Relief (PEPFAR) data from 2018 to 2019 reported an overall 3.6% positivity rate compared to rates of 15.4% and 21.1% in systematic reviews that included studies dating back to 2005.^2,3,9^ These studies did not disaggregate results by different wards, but it is programmatically important to know which services typically have the highest HIV prevalence to prioritise inpatient PITC efforts, especially given the barriers to achieving full coverage.

Paediatric surgical wards in SSA had a very high burden of HIV early in the epidemic, with positivity rates of 40% reported in studies from Malawi (2001–2004),^10^ and 55% in South Africa (2005).^11^ More recent research suggests a lower HIV burden without known changes in the indications for admission to surgical wards, with a study from Mozambique reporting a 7.1% PITC positivity rate in hospitalised children with surgical diagnoses, and another from a paediatric surgical ward in Zimbabwe reporting an HIV prevalence of 2.8%; however, both studies were limited due to testing coverage of less than 60% of eligible patients.^7,12^

## Methods

### Study context and design

A retrospective review of PITC, EID, and HIV serology data for paediatric surgical patients was performed.

### Sites

Hospital Central de Maputo (HCM), Hospital Central da Beira (HCB), and Hospital Central de Nampula (HCN) are the three largest referral hospitals in Mozambique, serving the southern, central, and northern regions of the country, respectively. All three hospitals have paediatric surgical services with inpatient wards. Both HCM and HCB have point-of-care EID, while HCN uses dried blood spot samples which are sent to a central laboratory on the same campus that offers conventional DNA polymerase chain reaction testing, with an average turn-around time of approximately 1 week.

### Population

All patients < 15 years of age admitted to the paediatric surgical wards of HCM, HCB and HCN between October 2019 and June 2020 were included. Aggregate monthly PITC data were systematically collected during this time period as part of a quality improvement project. Children requiring paediatric surgical consultation on other wards or in the outpatient setting were not included.

### Data collection

At the time of chart closure, patients’ basic demographic, diagnostic, and treatment data are documented in a ward discharge register. These registers were adapted to include basic PITC information, including HIV test results and information about ART when relevant. Aggregate monthly PITC data detailing the proportion of patients who had confirmed HIV serostatus at the time of inpatient chart closure were compiled by site surgeons or surgical residents. They collected additional clinical and demographic data from the ward discharge registers for HIV-exposed and positive children without patient identifiers. All data were entered into a Microsoft Excel^®^ database. Hospital charts were not used for data collection.

### Data analysis

Patient PITC coverage was determined by the number of patients whose HIV serostatus was known at discharge. Descriptive statistics (frequencies and medians), using Microsoft Excel^®^, were used to summarise PITC coverage, positivity, linkage to ART, and clinical profile of HIV-positive patients. Assessments about the relationship of the surgical diagnosis to HIV were made by study surgeons and paediatricians.

## Results

### PITC coverage

**FIGURE 1:**
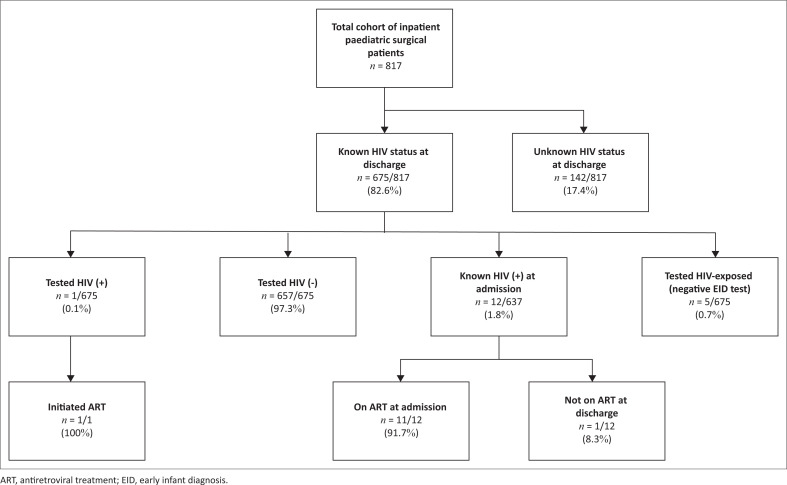
Provider-initiated testing and counselling and ART linkage results for children hospitalised on paediatric surgical wards in the three principal referral hospitals in Mozambique, October 2019 – June 2020.

A total of 817 patients were discharged from the three surgical wards during the time period of the study. The PITC coverage rate over the entire 9-month period was 84.6% at HCM (482/570), 77.6% at HCN (128/165), and 79.3% at HCB (65/82), for an overall coverage rate across the three hospitals of 82.6% (675/817) (Figure 1). Excluding March 2020, when there was significant disruption of work flows due to the onset of the coronavirus infectious disease (COVID-19) pandemic, the overall PITC coverage rate was 89.0% (607/682). Across the three hospitals, the monthly PITC coverage rate, defined as the percentage of eligible patients who were tested, increased from 72.2% (65/90) in October 2019 to 92.7% (38/41) in June 2020.

### PITC positivity, HIV prevalence, and ART linkage

Across all sites and throughout the entire study period, only one patient was newly diagnosed with HIV, giving a PITC positivity rate of 0.1% (1/675). An additional 12 patients were already known to be HIV positive at the time of admission, giving an HIV prevalence of 1.9% (13/675) in those with known serostatus and 1.6% (13/817) in the total cohort. At the time of discharge, 92.3% (12/13) of HIV-positive patients were on ART (Figure 1).

### Clinical profile of HIV-positive patients

The median age of HIV-positive patients was 7 years, and 38.5% (5/13) were female. HIV was deemed to be likely, possibly, unlikely or not related to the surgical diagnosis in 30.8% (4/13), 15.4% (2/13), 23.1% (3/13), and 30.8% (4/13) of patients, respectively (Table 1).

**TABLE 1:** Clinical profile of HIV-positive children admitted to paediatric surgical wards in the three principal referral hospitals in Mozambique, October 2019 – June 2020.

Age (years)	Sex	Diagnosis	HIV-related?†	Surgery performed
1	M	Inguinal abscess	Likely	Incision and drainage
3	F	Umbilical hernia	No	Hernioplasty
5	F	Inguinal hernia	No	Herniotomy
5	M	Scalp contusion	No	None
6	M	Pressure ulcer, paralysis	Likely	None
6	M	Pressure ulcer, sepsis	Likely	None
7	F	Abdominal rhabdomyosarcoma	Possibly	Biopsy
8	F	Clitoral oedema	Unlikely	None
10	M	Hirschsprung disease	No	None
10	M	Hodgkin lymphoma	Likely	Laparotomy and biopsy
11	M	Appendicitis	Unlikely	Appendectomy
12	F	Cellulitis	Possibly	None
14	M	Appendicitis	Unlikely	Appendectomy

M, male; F, female.

† , Assessed by study surgeons and paediatricians.

## Discussion

This study from the three largest referral hospitals in Mozambique had a relatively high testing coverage rate of 82.6% and an overall HIV prevalence of only 1.6%. Only one patient was newly diagnosed with HIV and the PITC positivity rate was 0.1%. This is in stark contrast to the PITC positivity rates as high as 55% reported from the period 2001–2005.^10,11^ Notably, 46% of HIV-positive patients in this cohort had diagnoses that were possibly or likely related to HIV, compared to 73% of HIV-positive children admitted to a surgical ward in South Africa from 2004 to 2006.^13^ Clearly, the huge toll of HIV that was previously reported in paediatric surgical patients has shifted dramatically over the last two decades with the scale-up of PMTCT, EID, and ART programmes.^8^

Given the very low prevalence of HIV seen in paediatric surgical patients in this study, a targeted testing approach could be considered for more efficient use of limited resources. A study from a paediatric ward in northern Malawi from 2016 to 2017 reported an HIV prevalence of 1.1% and a high negative predictive value for a screening tool comprising six questions about previous hospitalisations, parental deaths, and clinical conditions to identify at-risk patients for targeted testing; they recommended further research on this approach compared to routine PITC in low-prevalence settings.^14^ Although the HIV prevalence seen in this cohort was similarly low, another recent study from Mozambique conducted at smaller hospitals that do not have separate paediatric surgical wards reported a higher PITC positivity rate of 7.1% in children with surgical diagnoses.^7^ A 2022 South African modelling study reported that routine paediatric PITC is cost-effective when the prevalence of undiagnosed HIV is > 0.2%.^15^ If the true PITC positivity rate for hospitalised paediatric surgical patients in Mozambique is any higher than the 0.1% reported in this study, then routine PITC would likely be cost-effective.

There are also practical considerations that favour routine PITC for surgical patients. Determination of patient serostatus prior to operative procedures is best practice in high-prevalence settings in the case of sharps injuries. Implementing a mix of targeted PITC on paediatric surgical wards and routine PITC on other higher-yield wards would be difficult in terms of healthcare worker training, supervision, and monitoring and evaluation.

A recent analysis of PEPFAR testing data from 2018 to 2019 reported a paediatric ART coverage rate of 71.6%, a paediatric testing gap of 45.6%, and an estimated number needed to test for identification of one HIV-positive child from the inpatient setting of 36 for Mozambique – the second lowest result of the 16 countries included.^9^ Intensified routine inpatient PITC is clearly needed, but given the challenges and limited resources for implementation, the findings from this study can be used to help hospitals that have not yet achieved full testing coverage prioritise higher-yield paediatric patients.^4,5,6^ Critical care units and wards with children hospitalised with sepsis, tuberculosis and malnutrition will have higher PITC yields, as will wards for breastfeeding infants, given high rates of late-pregnancy and post-natal maternal seroconversion.^7,8^

This study had limitations. Children with surgical diagnoses hospitalised on non-surgical wards were not included, nor were children seen in outpatient surgical consultation, and there was a decline in PITC coverage at the onset of the COVID-19 pandemic in March 2020. Despite these limitations, this study had national representation with a high number of admissions and can be used to improve inpatient paediatric PITC programming, both nationally and in the SSA region.

## Conclusion

HIV prevalence and PITC positivity are currently much lower on paediatric surgical wards compared to reports from earlier in the HIV epidemic. Routine PITC is still recommended, but for paediatric hospitals struggling to achieve full inpatient PITC coverage, priority should be given to wards with a higher burden of HIV.

## References

[1] World Health Organization. Consolidated guidelines on HIV testing services, 2019. Geneva: World Health Organization; 2020.

[2] Cohn J, Whitehouse K, Tuttle J, Lueck K, Tran T. Paediatric HIV testing beyond the context of prevention of mother-to-child transmission: A systematic review and meta-analysis. Lancet HIV. 2016;3(10):e473–e481. 10.1016/S2352-3018(16)30050-9 27658876

[3] Govindasamy D, Ferrand RA, Wilmore SM, et al. Uptake and yield of HIV testing and counselling among children and adolescents in sub-Saharan Africa: A systematicreview. J Int AIDS Soc. 2015;18(1):20182. 10.7448/IAS.18.1.20182 26471265 PMC4607700

[4] Roura M, Watson-Jones D, Kahawita TM, Ferguson L, Ross DA. Provider-initiated testing and counselling programmes in sub-Saharan Africa: A systematic review of their operational implementation. AIDS. 2013;27(4):617–626. 10.1097/QAD.0b013e32835b7048 23364442

[5] Ahmed S, Schwarz M, Flick RJ, et al. Lost opportunities to identify and treat HIV-positive patients: Results from a baseline assessment of provider-initiated HIV testing and counselling (PITC) in Malawi. Trop Med Int Health. 2016;21(4):479–485. 10.1111/tmi.12671 26806378 PMC4881304

[6] Graça D, Elliott RJ, Magalo M, et al. Monitoring and evaluation of HIV screening and testing of hospitalized infants and their mothers. Public Health Action. 2022;12(2):68–73. 10.5588/pha.21.0074 35734006 PMC9176192

[7] Nhabomba C, Chicumbe S, Muquingue H, et al. Clinical and operational factors associated with low pediatric inpatient HIV testing coverage in Mozambique. Public Health Action. 2019;9(3):113–119. 10.5588/pha.19.0015 31803583 PMC6827495

[8] Chi BH, Mbori-Ngacha D, Essajee S, et al. Accelerating progress towards the elimination of mother-to-child transmission of HIV: A narrative review. J Int AIDS Soc. 2020;23(8):e25571. 10.1002/jia2.25571 32820609 PMC7440973

[9] Gross J, Medley A, Rivadeneira E, et al. Considerations to improve pediatric HIV testing and close the treatment gap in 16 African countries. Pediatr Infect Dis J. 2023;42(2):110–118. 10.1097/INF.0000000000003778 36638395 PMC10935587

[10] Masache E, Wilde J, Borgstein E. Indications for HIV testing in paediatric surgical patients. Malawi Med J. 2005;17(1):17–18. 10.4314/mmj.v17i1.10865 27528992 PMC3346036

[11] Bowley DM, Rogers TN, Meyers T, Pitcher G. Surgeons are failing to recognize children with HIV infection. J Pediatr Surg. 2007;42(2):431–434. 10.1016/j.jpedsurg.2006.10.016 17270563

[12] Migaud P, Silverman M, Thistle P. HIV status and mortality of surgical inpatients in rural Zimbabwe: A retrospective chart review. S Afr J HIV Med. 2019;20(1):812. 10.4102/sajhivmed.v20i1.812 PMC6407318 30863621

[13] Karpelowsky JS, Leva E, Kelley B, Numanoglu A, Rode H, Millar AJ. Outcomes of human immunodeficiency virus-infected and -exposed children undergoing surgery – A prospective study. J Pediatr Surg. 2009;44(4):681–687. 10.1016/j.jpedsurg.2008.08.036 19361626

[14] Moucheraud C, Chasweka D, Nyirenda M, Schooley A, Dovel K, Hoffman RM. Simple screening tool to help identify high-risk children for targeted HIV testing in Malawian inpatient wards. J Acquir Immune Defic Syndr. 2018;79(3):352–357. 10.1097/QAI.0000000000001804 29995704

[15] Stanic T, McCann N, Penazzato M, et al. Cost-effectiveness of routine provider-initiated testing and counseling for children with undiagnosed HIV in South Africa. Open Forum Infect Dis. 2022;9(1):ofab603. 10.1093/ofid/ofab603 35028333 PMC8753042

